# Visual Scanning in Very Young Children with Autism and Their Unaffected Parents

**DOI:** 10.1155/2012/748467

**Published:** 2012-03-26

**Authors:** Wouter B. Groen, Nanda Rommelse, Tessa de Wit, Marcel P. Zwiers, Desley van Meerendonck, Rutger Jan van der Gaag, Jan K. Buitelaar

**Affiliations:** ^1^Karakter, Child and Adolescent Psychiatry University Center, 6525GC Nijmegen, The Netherlands; ^2^Radboud University Nijmegen Medical Center, 6500HB Nijmegen, The Netherlands; ^3^Center for Cognitive Neuroimaging, Radboud University Nijmegen, 6525EN Nijmegen, The Netherlands

## Abstract

This study of gaze patterns in very young children with autism and their parents included 23 cases (with 16 fathers and 19 mothers) and 46 controls (with 14 fathers and 28 mothers). Children (mean age 3.3 ± 1.5 years) with autism met DSM-IV and ADOS-G diagnostic criteria. The participants' gaze patterns were recorded while they viewed four simple movies that did not feature people. In children, severity of autism is related to spending more time watching irrelevant regions in one of the four movies. The mothers of children with autism showed an atypical pattern for three movies, whereas the fathers of children with autism did not show an atypical gaze pattern. The gaze pattern of the mothers was positively correlated with that of their children. The atypical viewing pattern of autistic individuals appears not to be restricted to people and social situations but is also seen in other situations, suggesting that there is a perceptual broad autism phenotype.

## 1. Introduction

Autism spectrum disorders (ASDs) are a group of behaviorally defined disorders with impaired social interaction as a key feature, along with impairments in verbal and nonverbal communication and stereotyped and rigid patterns of behavior. There is evidence that these behavioral characteristics are accompanied by an atypical style of perception that is unique to autism [1–3]. Unlike individuals with other brain disorders, people with autism perform better than controls on tasks that involve the perception of low-level stimuli, such as discriminating visual luminance contrasts [2] and pure tones [3], but have a poorer performance on tasks involving complex stimuli [4]. The asymmetric perceptual pattern in autism has been explained using different but converging theoretical frameworks, such as the Weak Central Coherence Theory [5] and the Enhanced Perceptual Functioning model [6]. The main theme of these theoretical frameworks is that people with autism have difficulty (or are less inclined to) processing complex dynamic stimuli but are superior in processing simple static stimuli, leading to an atypical perceptual style. This atypical perceptual style may lead to difficulties in everyday life [7] if people with autism fail to identify and pay attention to relevant aspects of their environment. Failure to notice these stimuli could lead to different experiences and subsequently to different cognitive processes and behaviors during development [8], which in turn could lead to different perceptual styles, thereby forming a vicious cycle. Klin and colleagues argue that different perceptual preferences in early life lead to a self-amplifying developmental derailment in autism [8]. A recent eye-tracking study provided empirical evidence that perceptual styles change during development and differently in individuals with autism [9]. Given that autism has its roots very early in development [10], it is important to study perception in very young children.

 A number of studies have used eye tracking to investigate perceptual patterns in toddlers with autism (see Table 1); most studies investigated face processing [9, 11–16], but some investigated motion perception [16, 17]. Results suggest that children with autism tend to focus on the area around the mouth rather than on the socially informative eye area [14] and on static objects rather than (moving) people [16, 17]. Visual face processing appears to be affected early and becomes further compromised with age [11], which supports Klin and colleagues hypothesis of developmental derailment in autism [8, 17].

Eye tracking has also been used to study perceptual styles in the so-called broad autism phenotype. The broad autism phenotype includes subclinical impairments in language, communication, and social interaction that are found in unaffected family members of people with autism. Studies have shown that the broad autism phenotype is not limited to the triad of impairments but includes perceptual styles as well [18, 19]. For instance, gaze fixation and brain function in response to images of human faces were different in unaffected siblings and typically developing controls [20]; processing of the eye region in faces was reduced whereas that of the mouth region was increased in the parents of children with autism, but only in parents who were assessed as being socially aloof [21]; the 6-month-old siblings of children with autism spent less time looking at their mothers' eyes than did control siblings [22]. Taken together, these studies suggest that atypical perceptual styles may also be present in family members with milder or no autism traits, suggesting perceptual styles may be fruitful in the search for susceptibility genes for autism by acting as endophenotypes (heritable vulnerability traits that increase the liability to develop ASD) [23]. In this study, we used eye-tracking technology to determine whether visual scanning patterns are different in very young children with autism and their parents compared with normally developing children and their parents. We investigated very young children to establish whether perceptual style is different at a relatively early stage of developmental derailment and included parents to investigate whether atypical perceptual styles are familial.

## 2. Materials and Methods

### 2.1. Participants

This study of gaze patterns in very young children with autism and their parents included 23 cases (with 16 fathers and 19 mothers) and 46 controls (with 14 fathers and 28 mothers). Children with autism had been referred to the outpatient unit of Karakter Child and Adolescent Psychiatry University Center Nijmegen. Children were included in the autism group if they met criteria for autism on the ADOS-G, a standardized instrument administered directly to children [24], and DSM-IV diagnostic criteria for autistic disorder [25]. The DSM-IV criteria were established during a series of clinical assessments that included a detailed developmental history, clinical observation, medical work-up by a child psychiatrist, and cognitive testing by a clinical child psychologist. The ADOS-G was administered by an independent trained clinician who had not been involved in the diagnostic process. To exclude mental retardation, a clinical child psychologist assessed the cognitive development of the children with autism, using the Psychoeducational Profile Revised (PEP-R). The PEP-R is an inventory of behaviors and skills and is designed to identify uneven and idiosyncratic learning patterns and provides information on developmental functioning in imitation, perception, fine motor, gross motor, eye-hand integration, cognitive performance, and cognitive verbal areas [26]. Control participants (children and their parents) were recruited from local daycare centers. To exclude psychiatric disorders or learning problems, parents completed the CBCL questionnaire [27]. None of the control participants had scores on the CBCL in the clinical range. To exclude mental retardation, the cognitive development of the control children was assessed with the Mullen Scales of Early Learning [28].

 The parents of control children and children with autism completed the Autism Spectrum Quotient questionnaire (AQ), which evaluates the presence of mild autistic traits in adults of normal intelligence (including social skills, attention switching, attention to detail, communication, and imagination) [29]. The AQ is a 50-item 4-point Likert scale in which incremental scores (ranging from 0 to 200) for correlation analysis or diagnostic scores (ranging from 0 to 50) for diagnostic purposes can be calculated (note that in Table 2 incremental scores are used). None of the parents had been diagnosed with an ASD, and only one parent (a father of a child with autism) scored above 32 on the AQ on the diagnostic scheme (which is a commonly used threshold for autism as the probability of someone without ASD, obtaining a score above 32 is just 2% [29]). Exclusion criteria for all participants were mental retardation, any general medical condition affecting brain function, neurologic disorders, and substance abuse. The study was approved by the local medical ethics committee. Informed consent was obtained from all parents.

### 2.2. Procedure

The participating children were invited to play with their parents and the third author (TW) for several minutes in the light-shielded, child-tailored research laboratory of Karakter Child and Adolescent Psychiatry University Center, so that they could become accustomed to the room and the Tobii Eye-tracking device. The third author (TW) then invited the child to “watch TV” on the 17-inch Tobii monitor, which resembled an ordinary flat-screen TV; the child sat in a child's chair. A 5-point eye-tracking calibration procedure was initiated. The calibration was repeated if necessary until all 5 calibration points were properly identified. The parents waited in another room, and the child then watched the four movies with only the third author present during the 15-minute period. After calibration, the parents then watched the same movies, again with only the third author present.

 All participants sat 70 cm from the 1024 by 1280 pixel “landscape” monitor, in which the eye-tracking technology is invisibly integrated. Using infrared light, the Tobii T120 (Stockholm, Sweden) tracks pupil movements and size at 60 Hz with a spatial resolution of 0.5° using corneal reflection patterns. Thus, high-precision measurements could be made while participants were free to move (within a virtual cubic space of 44 × 22 × 30 cm) and were not influenced by data acquisition through physical contact or feedback. Eye-tracking patterns (2-dimensional fixation coordinates of the left and right eye and pupil size) were recorded on line and later analyzed using Matlab 7.5 (MathWorks, Natick, MA). This allowed us to calculate the percentage of time per movie that participants looked at specific regions of interest. The spatial and temporal coordinates of the regions of interest were extracted using Clearview, Tobii's stimulus presentation software. The regions of interest differed for each movie and contained the elements essential for comprehension of the movie.

All participants (children and parents) watched the same four movies, which were taken from popular children's TV shows. The movies were presented in a randomized order to counterbalance attention or learning effects. Importantly, parents and children were not instructed to attend to certain features in the movies, so that the watching paradigm provided a naturalistic instruction-free situation. This allowed us to measure tendencies rather than abilities, because this setup resembles daily life more closely than explicit tests.

### 2.3. Materials

The four movies were selected because they were easy to understand but provided a substantial amount of visual information; because they provided variable degrees of animation to provide for dynamic complex stimuli; because they did not contain actual people, as children with autism may actively avoid eye contact [30]. For all movies, the absolute and relative time participants spent watching regions of interest (ROIs) was calculated. Absolute time summed for all ROIs of a movie was calculated as Σ ((time tracked by the eye-tracker in the ROI/total time the ROI was visible) × 100%). Relative time summed for all ROIs of a movie was calculated as Σ ((time tracked by the eye-tracker in the ROI/total time the ROI was visible) × (100%/% of total time watched at the movie)). The ROIs were shaped in the form of a rectangle over the objects of interest. The position (on screen coordinates) and time window (time of onset and end time in ms) of the ROIs were obtained using Clearview (version 2.7.1); for moving ROIs, consecutive coordinates were obtained. Matlab (version 7.1) was used to calculate watching times within the ROIs, and SPSS (version 17) was for statistical testing.

**Figure 1:**
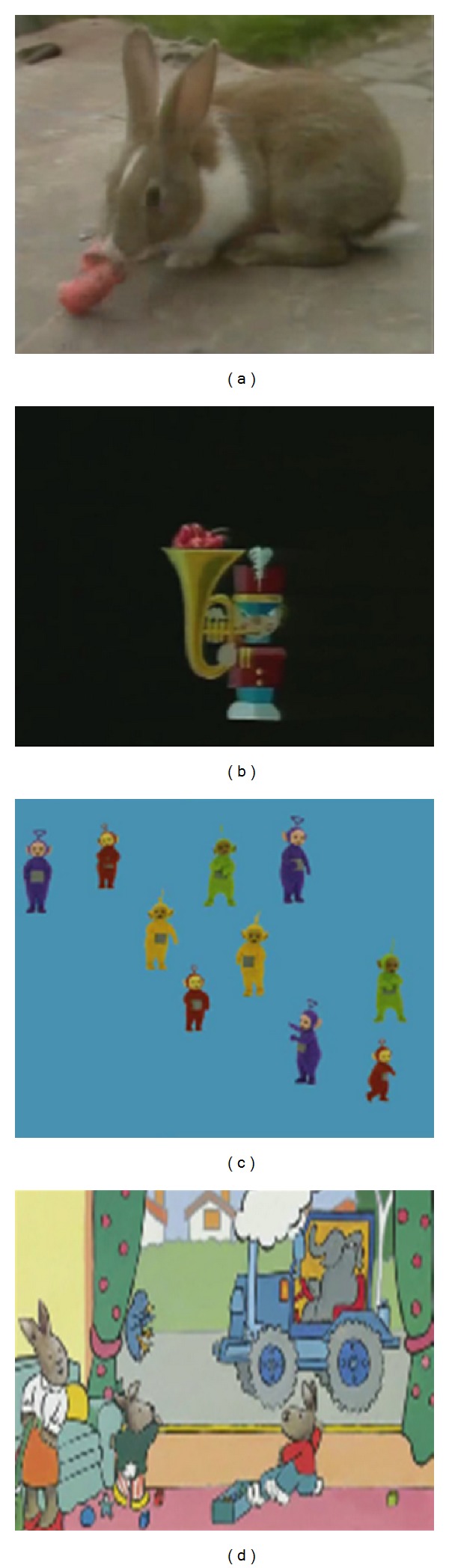
Clockwise, starting upper left: Puzzle Rabbit movie, Trumpet movie, Grandma Rabbit movie, and Teletubbies movie.

The Rabbit puzzle movie (duration: 33 s) showed nine puzzle pieces that moved from the left and right side into the screen to form a photorealistic picture of a rabbit with a carrot (Figure 1). When the ninth piece moved into place at *t* = 24 s, the picture unfroze and showed a rabbit eating the carrot. ROIs were the eye and the ears of the rabbit and the carrot. As the ears and the eye were visible from *t* = 10–33 s, their corresponding ROIs started at *t* = 10 s and ended at *t* = 33 s. The carrot ROI started at *t* = 15 s and ended at *t* = 33 s. As the picture of the rabbit unfroze at *t* = 24 s, the ROIs positions moved to cover the eye, ear, and carrot from *t* = 24 to *t* = 33.

 The movie Trumpet (duration: 26 s) featured a single puppet that moved from left to right and back while playing on his trumpet (Figure 1). At the end of the movie (at *t* = 21 s), the puppet's position was static and it blew its trumpet: a large purple flower popped out of the trumpet. ROIs were the flower and face. The position of the face ROI moved horizontally from *t* = 0 to *t* = 21 to follow to puppet's movements and remained static from *t* = 21–26 s. The flower ROI was present from *t* = 21–26 s.

 The Teletubby movie (duration: 29 s) showed 17 Teletubbies that subsequently appeared every few seconds and filled the screen (Figure 1). They moved as they stood, but they did not change their position on the screen. As several Teletubbies kept popping up, viewers do not usually spot newly appearing Teletubbies. Rather, people tend to pay attention to the central Teletubbies, to examine their appearance. The three central Teletubbies were combined into 1 ROI that was present from *t* = 4 s to *t* = 29 s. Also, the number of newly appearing targets spotted was calculated. Teletubbies could be spotted from the moment they appeared until a new Teletubby appeared.

 The Grandma Rabbit movie (duration: 1 min 26 s) consisted of eight drawings that showed a mother rabbit with her four children in their house (Figure 1). At *t* = 0 s, 10 s, 24 s, 37 s, 51 s, 63 s, 74 s, and 83 s, a new drawing appeared. In every drawing but the last, interesting objects such as a school bus or an ice-cream van could be seen through the window. In the last drawing, Grandma Rabbit could be seen through the window riding her motorbike. ROIs were the rabbits' faces and the window. The ROIs were present from *t* = 0–80 s. The ROIs were static most of the time, but as the transitions between drawings involved a limited amount of panning and zooming, the ROIs moved to follow the faces and the window during the transitions.

### 2.4. Data Analyses

Eye-tracking data were available for 22 of 23 children, 10 of 16 fathers, and 16 of 19 mothers from ASD families, and for 46 of 46 children, 12 of 14 fathers, and 26 of 28 mothers from control families. A minimum of 50% valid gaze time was required for analysis. Subjects were excluded if they had no valid data on any of the four movies, for example, due to excessive subject movement. Skewness and kurtosis were examined for each variable to test for normal distribution. To examine whether the facial fixation patterns of the children with autism and their fathers and mothers differed from those of the control children and their fathers and mothers, respectively, one-sided independent samples *t*-tests were performed with the percentage of absolute and relative time spent watching ROIs as dependent variables. If significant group differences were found between the ASD and control children, correlations were calculated between ADOS scores and the time spent watching the ROIs for the group of children with autism, to examine whether abnormal watching behavior was related to the severity of autism. Thereafter, to examine the relationship between parental and offspring watching behavior, generalized estimation equations (GEEs) were used with an exchangeable working correlation matrix, scale parameter method on deviance, and robust estimators. Family number was used as subject effect to account for clustered data (e.g., in several control families multiple children participated, resulting in clustered parent-offspring pairs). Independent variables were percentage of absolute time watching the ROIs for fathers and mothers separately. Diagnosis (ASD versus control) and sex of the child were also added as predictors. The percentage time the child spent watching the ROIs was dependent variable. Analyses were repeated for relative watching time. Analyses were carried out in SPSS version 17. For all analyses, correction for multiple testing using the 95% CI was performed, using the false discovery rate procedure.

## 3. Results and Discussion

### 3.1. Results

There were no significant age or sex differences between the ASD and control families (see Table 2). The fathers, but not the mothers, of the children with autism reported slightly more problems on the imagination and the total scales of the AQ than did control fathers.

 Skewness and kurtosis were acceptably low for all variables. Independent sample *t*-tests (Table 3) indicated that the children with autism spent less time than the control children watching the ROIs in the Grandma Rabbit movie, although this finding did not survive stringent correction for multiple testing. However, correlation analyses revealed medium to large correlations between watching parameters of the Grandma Rabbit movie and several ADOS scores (absolute time watching ROIs: play *r* ^2^ = −0.47, *P* = 0.05; relative time watching ROIs: play *r* ^2^ = −0.49, *P* = 0.04; social *r* ^2^ = −0.52, *P* = 0.02; time watching the movie: play *r* ^2^ = −0.44, *P* = 0.05), indicating that children with more severe autism (higher ADOS scores) spent less time watching the ROIs and the movie in general. The mothers of children with autism showed abnormal watching behavior, spending less time than control mothers watching the ROIs on three of the four movies (Puzzle Rabbit, Grandma Rabbit, and Teletubbies). These findings were still significant after correction for total watching time. The fathers from ASD families did not differ from the control fathers in their watching behavior. No group differences were found in the number of newly spotted Teletubbies in ASD versus controls, respectively (children *M* = 9.0 and *M* = 9.0, *P* = 0.49; fathers *M* = 11.3 and *M* = 13.4, *P* = 0.09; mothers *M* = 10.4 and *M* = 12.1, *P* = 0.13).

GEE analyses were performed to investigate the relatedness of parent-offspring watching behavior. These analyses were performed for mother-child watching behavior (52 mother-child pairs were available for analyses) but not for father-child watching behavior, because too few father-child pairs were available for analysis (only 26 father-child pairs were available) and because the fathers of children with autism did not show abnormal watching behavior. The time (relative and absolute) mothers spent watching ROIs in the Teletubbies movie was positively correlated with the corresponding measures in their offspring (*Wald *χ*²* = 3.53, *P* = 0.05 and *Wald *χ*²* = 6.45, *P* = 0.01, resp.). No significant effects were found for the other movies.

### 3.2. Discussion

In the present study, we investigated free visual scanning in a large sample of very young (about 3 years of age) children with autism and their parents. As perceptual tendencies drive behaviors and behaviors drive perceptual tendencies, a circular process of developmental derailment may ensue in autism [8]. We studied very young child to investigate when this putative derailment occurs. We found subtle visual scanning differences between children with autism and control children for one of the four movies. Within the group of children with autism, higher ADOS scores related to more abnormal watching behavior. While the perceptual style of the fathers of children with autism was not different from that of the control fathers, the perceptual style of the mothers of children with autism was in the main atypical. These mothers spent less time than the control mothers watching the most relevant parts of the videos even after correction for the total watching time, suggesting that their attention and perceptual styles were different from those of control mothers. Lastly, direct parent-offspring associations in watching behavior showed that the children with autism and their mothers had similar gaze patterns, although this relation was significant for only one movie (Teletubbies), perhaps due to the limited sample size.

 The results suggest that the atypical perceptual style of autistic individuals and the perceptual broad autism phenotype are not restricted solely to the social domain, as the children with autisms paid less attention to relevant aspects of visual, nonhuman, stimuli. As people with autism may fail to identify and pay attention to relevant aspects of their environment, differences in perceptual tendencies may in part explain the often-observed discrepancy between their good performance on formal social-cognitive tests and their difficulties in everyday life [7, 31]. In an elegant study, Neumann and colleagues investigated which processes drive the abnormal perceptual style in people with autism [32]. Using eye tracking and faces with varying contrasts and intensities, they were able to model the contribution of bottom-up and top-down processes that drive eye gaze in autism. That is, simple features, such as high contrasts or motion, influence eye movements in a bottom-up fashion, while top-down modulation is based on stimulus meaning, learned associations, and expectations [32]. As they found only a low correlation between low-level visual saliency and gaze patterns, Neumann et al. argued that atypical perception in autism is mainly driven by an abnormal top-down strategy for allocating visual attention.

 Interestingly, the notion that mainly top-down processes, such as learned associations and expectations, drive the perceptual style in autism is consistent with the hypothesis of self-amplifying developmental derailment in autism, in which early atypical perceptual styles give rise to more atypical perceptual styles in later life. Low-level saliency is less likely to change during development than are top-down processes, because top-down processes are the product of learned associations. Of note, a study of temporospatial gaze patterns in children and adults with and without autism, using multidimensional scaling [9], showed that children's and adults' temporospatial gaze patterns clustered differently, indicating that gaze behaviors developed or changed over time. Post hoc analyses revealed that typical children preferred to watch the mouth rather than the eyes during speech, while adults preferred to watch the eyes rather than the mouth. This difference was not seen in children and adults with autism. Longitudinal studies of child-parent watching behavior would add a wealth of empirical data to the developmental derailment hypothesis. One would expect that the mothers who exhibited the most atypical watching behavior would have children whose watching behavior would develop atypically over time. The observation that the children with autism and their mothers showed atypical perceptual patterns provides some empirical evidence for the hypothesis of developmental derailment in autism. However, as the parents did not have an ASD, the current study design does not allow for solid inferences on perceptual styles in adults with autism.

 Another important implication of the current findings is that while the parents of children with autism were not impaired clinically, the mothers showed perceptual patterns similar to those seen in individuals with autism. This suggests that atypical perceptual patterns are an endophenotypic trait. The current findings therefore validate the concept of the perceptual broad autism phenotype [19] and underline the usefulness of this endophenotypic trait for brain and genetic studies.

 The eye-tracking data furthermore suggest that the broader autism phenotype not only applies to siblings [20] but to mothers as well. This is important since it is often suggested that the broad autism phenotype is found in the fathers but not the mothers of children with autism [33]. It is remarkable that the mothers' visual scan patterns were affected the most, since their AQ scores did not differ from the scores of the control mothers. The fathers of children with autism, on the other hand, ascribed more autistic traits to themselves in the total AQ score and the imagination subscore specifically. Only one other study has previously used the AQ with the parents of children with autism [33]. The authors also reported that the mothers of children with autism had lower AQ scores than the control parents, which the authors interpreted as showing that the mothers of children with autism are reluctant to ascribe autistic traits to themselves. Self-report questionnaires of autistic traits may thus underestimate autistic traits in mothers. Given our results of impaired perceptual styles in these self-reported unimpaired mothers, it seems vital to include non-self-report measures of autistic traits in mothers of children with autism when studying the familiality of autism.

Some limitations need to be taken into account. First, the cognitive development of the children with autism and the control children was assessed using different tests, which prevents direct comparison of the children's cognitive skills. Second, although the total number of participants was relatively large for an eye-tracking study, the number of parent-offspring pairs and the size of the autism sample overall were relatively limited, reducing statistical power. Third, alternative explanations for the gaze pattern differences between the mothers of children with autism and the control mothers cannot be ruled out. Having a child with autism may cause differences in eye gaze patterns as a result of another process such as stress. As the mothers of autistic children may experience more stress than the fathers, our finding of differences between mothers but not fathers might be due to greater levels of maternal stress. This explanation is, however, not in line with findings of increased perceptual abilities in people with autism that cannot be accounted for by greater stress [2].

## 4. Conclusions

The current findings suggest that the atypical perceptual style in autism is not solely limited to the social domain and validate the concept of a perceptual broad autism phenotype. The results are in line with the hypothesis of developmental derailment, in which early atypical perceptual styles give rise to more atypical perceptual styles in later life. Converging evidence suggests that atypical perceptual patterns reflect the developmental unfolding of selective learning profiles in children with autism [17]. Combining the perceptual endophenotype with genetic studies may shed light on the genetic and neurobiological anomalies in autism.

## Figures and Tables

**Table 1:** Overview of recent influential eye-tracking studies in very young children.

Study	Age (y)	N (a–c)	Method	Main results and conclusions
Chawarska and Shic 2009 [11]	2–4	44–30	Visual scanning and recognition of faces	R: children with autism looked increasingly away from faces with age and atypically attended to key features of faces C: face processing is affected early and becomes further compromised with age
Chawarska et al. 2010 [12]	2–4	42–46	Attentional bias associated with faces and nonfacial stimuli	R: controls had more difficulties disengaging visual attention from faces but not objects than children with autism C: the neural attentional mechanism that supports deep processing of faces is disrupted in autism
Falck-Ytter et al. 2010 [13]	4–6	15-15	Visual scanning of faces	R: children with autism who are better at socioemotional skills than nonverbal communication skills look more at the eyes than the mouth, and vice versa C: separate neural systems underlie these skills
Jones et al. 2008 [14]	2	15–36	Visual scanning of an actress playing the role of caregiver	R: looking at the eyes of others was decreased in children with autism, while looking at mouths was increased C: looking at the eyes is derailed early offering a potential biomarker quantifying syndrome manifestation
Klin and Jones 2008 [16]	1	1–0	Visual scanning of naturalistic and ambiguous social stimuli	R: viewing patterns of a child with autism were driven by the physical contingencies of the stimuli rather than by their social context C: mechanisms of social development are developmentally derailed in children with autism
Klin et al. 2009 [17]	1–3	21–39	Visual scanning of point-light (inverted) displays of biological motion	R: children with autism fail to orient towards point-light displays of biological motion C: early developmental derailment leads to an altered neurodevelopmental trajectory of brain specialization in autism
Nakano et al. 2010 [9]	2–9; >25	25-25 27-27	Temporospatial gaze patterns of visual scanning of video clips	R: typical infants preferred to watch the mouth rather than the eyes, which reversed with development (eyes rather than mouth) C: research in gaze behavior should take the effect of development into account
Young et al. 2009 [15]	0.5	33–25	Live interaction with video-transmitted mothers' face	R and C: eye contact did not predict autism at follow up; greater amounts attention to the mother's mouth predicted higher levels of expressive language at follow up

a: autism spectrum disorder group; c: controls; C: conclusions; R: results.

**Table 2:** Participant characteristics.

	Children			Fathers			Mothers		
	Autism *N* = 23	Control *N* = 46	*t*/**χ*², P *	Autism *N* = 16	Control *N* = 14	*t, P *	Autism *N* = 19	Control *N* = 28	*t, P*
Age in years	3.1 (1.0–5.2)	3.6 (1.1–6.8)	1.34, .19	37.7 (30.8–48.1)	40.8 (35.4–47.3)	−1.48, 0.15	34.3 (27.1–40.1)	36.5 (31.4–46.0)	1.70, 0.10
Sex (*N*, %♂)	16 (72.7)	26 (56.5)	1.10, .30						

	ADOS								

Communication	22.3 (10.4)								
Social reciprocal interaction	10.7 (4.4)								
Play	3.2 (1.8)								
Stereotyped behaviors and restricted interests	3.1 (2.0)								

				Autism Spectrum Quotient					

Social skills				20.9 (7.7)	16.8 (3.4)	1.72, 0.10	17.0 (5.1)	15.8 (4.9)	0.71, 0.48
Attention switching				24.6 (8.2)	20.1 (4.3)	1.50, 0.15	17.4 (5.1)	19.4 (4.4)	−1.31, 0.20
Attention to detail				23.5 (7.7)	22.6 (3.6)	0.40, 0.70	19.7 (6.8)	22.8 (4.2)	−1.73, 0.09
Communication				20.4 (6.4)	17.0 (3.8)	1.64, 0.12	16.1 (3.6)	16.9 (4.4)	−0.62, 0.54
Imagination				22.9 (5.8)	18.1 (2.7)	**2.71,** **0.01**	15.2 (4.1)	17.6 (3.8)	−1.88, 0.07

Total				115.9 (31.2)	94.0 (9.0)	**2.40,** **0.03**	83.0 (19.3)	92.6 (14.8)	−1.67, 0.11

		Mullen T-scores							

Expressive language		55.4 (9.7)							
Receptive language		50.8 (6.7)							
Fine motor		50.0 (10.9)							
Visual reception		57.0 (9.5)							

	PEP-R								

Imitation	20.1 (6.3)								
Perception	33.8 (12.4)								
Fine motor	25.6 (7.0)								
Gross motor	25.1 (7.6)								
Eye-hand integration	26.6 (4.9)								
Cognitive performance	14.4 (5.4)								
Cognitive verbal	16.1 (7.9)								

**Table 3:** Percentage of absolute and relative time watched at regions of interest (ROIs) in children with ASD and control children and their fathers and mothers.

		Absolute time in %							Relative time in %						
		Autism		Controls					Autism		Controls				Orientation to ROIs
	Range	*M*	*SD*	*M*	*SD*	*P*	*d*	Range	*M*	*SD*	*M*	*SD*	*P*	*d*	
Children		*N* = 22		*N* = 46											

Movie Puzzle Rabbit	0–61	22.4	17.8	26.8	15.3	ns	0.3	0–66	26.2	18.4	31.1	15.6	ns	0.3	
Movie Trumpet	0–70	32.5	22.8	24.5	22.1	ns	0.3	0–100	39.0	28.4	31.0	27.9	ns	0.3	
Movie Teletubbies	0–47	18.3	8.4	21.2	12.6	ns	0.2	0–57	24.6	9.5	26.3	12.5	ns	0.2	
Movie Grandma Rabbit	1–72	32.1	20.7	42.8	19.1	0.02	0.5	10–100	45.4	17.5	54.8	20.0	0.04	0.5	Autism ↓ Controls

Mothers		*N* = 16		*N* = 26											

Movie Puzzle Rabbit	0–56	**19.1**	**11.6**	**30.3**	**13.4**	**<0.01**	0.9	0–58	**24.0**	**13.2**	**32.9**	**13.8**	**0.02**	0.7	Autism ↓ Controls
Movie Trumpet	0–84	32.5	21.8	44.3	26.1	ns	0.5	0–88	40.0	22.5	46.7	26.5	ns	0.3	Autism ↓ Controls
Movie Teletubbies	0–71	**19.3**	**13.2**	**28.8**	**13.5**	**0.02**	0.7	0–72	25.7	12.6	31.5	13.0	ns	0.4	Autism ↓ Controls
Movie Grandma Rabbit	0–69	**31.5**	**21.0**	**53.5**	**11.6**	**<0.001**	1.4	3–88	**50.3**	**18.7**	**61.0**	**11.0**	**0.01**	0.7	Autism ↓ Controls

Fathers		*N* = 10		*N* = 12											

Movie Puzzle Rabbit	0–53	27.8	15.3	30.5	11.2	ns	0.2	2–56	32.7	14.3	36.1	12.7	ns	0.3	
Movie Trumpet	3–69	27.7	20.7	33.0	18.8	ns	0.3	7–71	33.0	20.1	34.5	18.7	ns	0.1	
Movie Teletubbies	4–49	22.0	11.4	25.1	11.3	ns	0.3	10–52	27.8	10.6	27.3	10.9	ns	0.05	
Movie Grandma Rabbit	6–64	44.2	18.3	48.8	10.6	ns	0.3	26–98	60.9	17.9	56.5	6.7	ns	0.4	

Groups compared with one-sided independent samples *t*-tests. Ns: not significant. Findings printed in bold were significant after correction for multiple testing.

## Abbreviations

ASD: Autism spectrum disorders
AQ: Autism Spectrum Quotient questionnaire
ROIs: Regions of interest.
